# Lend Me Your EARs: A Systematic Review of the Broad Functions of EAR Motif-Containing Transcriptional Repressors in Plants

**DOI:** 10.3390/genes14020270

**Published:** 2023-01-20

**Authors:** Vanessa Chow, Morgan W. Kirzinger, Sateesh Kagale

**Affiliations:** Aquatic and Crop Resource Development, National Research Council Canada, Saskatoon, SK S7N 0W9, Canada

**Keywords:** transcriptional repression, EAR motif, plant growth, homeostasis, abiotic stress, plant immunity, plant reproduction

## Abstract

The ethylene-responsive element binding factor-associated amphiphilic repression (EAR) motif, defined by the consensus sequence patterns LxLxL or DLNx(x)P, is found in a diverse range of plant species. It is the most predominant form of active transcriptional repression motif identified so far in plants. Despite its small size (5 to 6 amino acids), the EAR motif is primarily involved in the negative regulation of developmental, physiological and metabolic functions in response to abiotic and biotic stresses. Through an extensive literature review, we identified 119 genes belonging to 23 different plant species that contain an EAR motif and function as negative regulators of gene expression in various biological processes, including plant growth and morphology, metabolism and homeostasis, abiotic stress response, biotic stress response, hormonal pathways and signalling, fertility, and ripening. Positive gene regulation and transcriptional activation are studied extensively, but there remains much more to be discovered about negative gene regulation and the role it plays in plant development, health, and reproduction. This review aims to fill the knowledge gap and provide insights into the role that the EAR motif plays in negative gene regulation, and provoke further research on other protein motifs specific to repressors.

## 1. Introduction

Transcriptional repression is a key regulatory mechanism essential for the modulation of gene expression during plant development, stress responses, and hormone signaling. In the past decade, remarkable progress has been made in elucidating the molecular nature and functions of transcriptional repression complexes [1]. Plants employ a wide repertoire of transcriptional repression mechanisms that are generally orchestrated by a complex and coordinated network of active or passive repressors, corepressors, components of basal transcriptional machinery and chromatin modifiers [2,3]. Active transcriptional repressors generally associate with target genes in one of two ways: by directly binding to their promoter elements through a DNA binding domain or indirectly by interacting with DNA-bound proteins, conferring repression. The latter is facilitated by either inhibiting the components of the basal transcriptional machinery or by recruiting chromatin modifiers which can modify chromatin structure and prevent transcriptional activators from binding to the target cis-elements [4,5].

The ethylene-responsive element binding factor-associated amphiphilic repression (EAR) motif-mediated transcriptional repression has emerged as one of the principal mechanisms for active repression of gene expression in plants. The EAR motif was first identified 20 years ago in plants [6] and its role in negative gene regulation has been well documented. Though more than 20,000 EAR motif-containing proteins exist in different plant species [7], 119 EAR motif-containing proteins have been functionally characterized to-date and the role of EAR motif in these candidates has been confirmed (Table 1). In this review, we focus on the similarities and differences between the fully characterized EAR repressors and leverage this information to provide insights into how the EAR motifs play an important role in negative regulation of gene expression in plants. As EAR motif-containing proteins have been characterized in a wide array of plants, this allows for comparisons and analyses to be conducted over a broader dataset of plant species. As such, in order to effectively analyze the EAR motif-containing proteins across an array of plant species, we separated the proteins into 7 categories according to known biological function (discussed in Section 3: Molecular functions of EAR motif-containing proteins). By classifying these proteins in this way, we were able to compare EAR motif-containing proteins that have similar functions across distinct species.

## 2. Consensus Sequence and the Localization of the EAR Motif

### 2.1. Consensus Sequence of the EAR Motif

**Figure 1:**
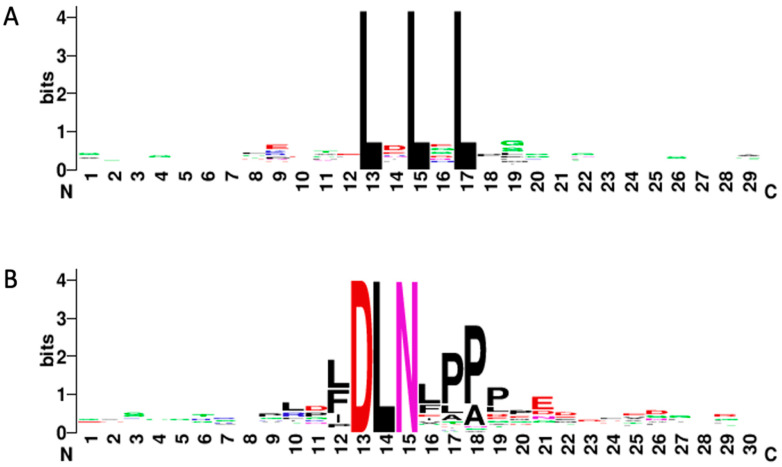
EAR motif sequence logos. (**A**) Sequence logo of the LxLxL EAR motifs including 12 amino acids upstream and 12 amino acids downstream; (**B**) Sequence logo of the DLNx(x)P EAR motifs including 12 amino acids upstream and 12 amino acids downstream.

The 119 functionally characterized EAR repressors contain a total of 143 EAR motif instances (Table S1) of which 45 (31.5%) contain DLNx(x)P type of EAR motif (Table 1, Tables S2–S5), and 110 (76.9%) contain a conserved consensus sequence of LxLxL (Tables S6–S8). Interestingly, 22 (15.5%) of the EAR motifs contained overlapping motif sequences—either multiple overlapping LxLxL motifs (14) or LxLxL motifs overlapping with the DLNx(x)P motifs (12). Through an analysis of the LxLxL motifs and the generation of the LxLxL sequence logo with 12 amino acids before and after the EAR motif (Figure 1A), it was discovered that there are several extended LxLxL motifs among the EAR repressors identified in this study. A total of 13 LxLxLxL EAR motifs were identified, including 4 LxLxLxL motifs that overlap with DLNx(x)P (Table S7). These motifs were identified in 4 different species and across 6 of the 7 biological functions, and most of them were identified in the C-terminal region of their respective proteins. We also identified two LxLxLxLxL motifs among the 119 proteins surveyed in this study: LVLLLVLFL and LELSLGLSL (Table S8). They were identified in different species, they are at opposite ends of their respective proteins, and though they are both in proteins with 2 different biological functions, there is no overlap between those functions. Furthermore, one protein is small (119 amino acids) and the other is longer in comparison (476 amino acids).

We can hypothesize that these extended LxLxL motifs are evolutionarily favorable for (1) broadening the scope of co-repressors that these proteins can bind to, and (2) providing stability to the LxLxL EAR motif should missense mutations occur over time. It would be advantageous to have multiple LxLxL motifs overlapping if the EAR motif binding sequence varies from co-repressor to co-repressor. This could provide a broader range of transcriptional repression through the recruitment of additional co-repressors. In the latter case, the extension of the LxLxL motifs could act as a failsafe in the event of genetic mutations that change the EAR motif sequence. This redundancy would help ensure that transcriptional repression still occurs even if there are changes to the extended LxLxL motif.

Furthermore, there are several instances where multiple EAR motifs were identified in the same protein. Of the 119 proteins analyzed in this survey, 24 were found to have two EAR motifs and 12 of those proteins had one overlapping EAR motif. For example, the IAA7 EAR repressor (an auxin-related protein that plays a role in development) in *Arabidopsis thaliana* contains two EAR motifs [8]. The second EAR motif in IAA7 was found to have a minor function compared to the first EAR motif; there was a difference in the co-repressors each one interacted with. As such, it may be evolutionarily advantageous to have more than one EAR motif if the protein interacts with co-repressors that differ in their chemical composition and therefore require specificity in the motifs that recruit them.

### 2.2. Significance of Proline and Its Location in Relation to the DLN Sub Motif in DLNx(x)P Type EAR Motifs

Liu et al. (2018) showed that the N-terminal DLNVESP EAR motif in the Q repressor in *Triticum aestivum* is responsible for mediating interaction with TOPLESS, but not the C-terminal LDLDL EAR motif [9]. This also raises an interesting point regarding the sequence of the DLNx(x)P EAR motif. The authors describe the N-terminal motif as LDLNVE; however, the sequence can be extended to LDLNVESP or more generally DLNxxxP, which is a variation of the DLNx(x)P motif. The presence of an additional amino acid between the DLN sub motif and the proline suggests that there could be some flexibility in the position of the proline at the end of the DLNx(x)P motif. This also could suggest that proline is required for proper binding of co-repressors and transcriptional negative regulation in EAR motifs containing DLN.

Among the 119 EAR motif-containing proteins surveyed in this study, we analyzed 12 bases upstream and 12 bases downstream of all DLNx(x)P motifs to generate sequence logos (Figure 1B), which revealed several instances where there was a proline in the 4th amino acid position away from the DLN sub motif (e.g., DLNxxxP) (Table S4). There are in fact 21 instances of a proline in this amino acid position. Nineteen of these motifs have already been included in this study (Table 2, Table S1); however, two new motifs were identified through this investigation: DLNMNLP (SlERF4-11) [10] and DLNVESP, which we discuss above (Q) [9]. It is worth noting that 12 of the 21 EAR motifs that have proline in the 4th amino acid spot after the DLN sub motif contain a DLNxPPP motif pattern, and 5 of these DLNxPPP motifs are DLNFPPP while another 4 are DLNLPPP. Among the remaining DLNxxxP motifs, there are two interesting patterns that surfaced with alanine and leucine amino acids: DLNLPAP and DLNLAPP both were identified 3 times among the 21 DLNxxxP EAR motifs, and DLNLPPP appears once. Though a more thorough analysis would be involved, we can only postulate that there is some significance to the higher presence of leucine, alanine, and phenylalanine in the DLNx(x)P EAR motif.

To build on the above inquiry, we looked further into our data to see if there was any indication that the proline could be further away from DLN than 3 amino acids. We evaluated the identified DLN motif sequences surveyed in the manuscript and looked for DLN sequences that contained proline in the 5th amino acid position after DLN. In total, 10 instances of DLNxxxxP were identified, all of which were previously identified EAR motifs (DLNx(x)P) (Table S5). Five of these motifs were DLNxP sub motifs containing the consensus motif DLNxPxxP, while the other five motifs were DLNxxP containing the consensus motif DLNxxPxP. These findings suggest that proline in the 3rd, 4th, or 5th position after DLN could potentially be involved in co-repressor binding, though in vitro experimentation would be required to confirm that this is the case. While it is also very difficult to draw conclusions based on such a small sample size, this analysis highlights the need for additional research in order to expand our understanding of the nature and scope that the EAR motif plays in negative gene regulation in plants.

### 2.3. Localization of the EAR Motif

**Figure 2:**
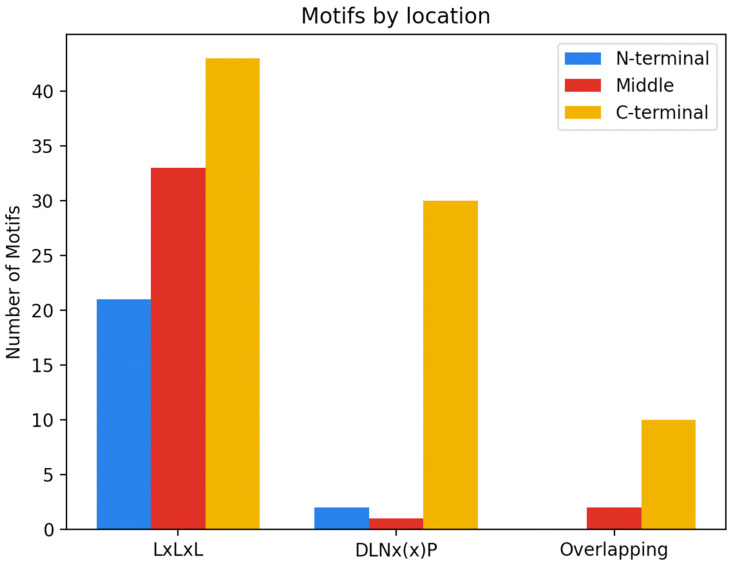
Distribution of EAR motifs. Box plots showing the number of EAR motifs based on the protein region in which they are found (N-terminal, middle, C-terminal) and sub-divided by the type of EAR motif: LxLxL, DLNx(x)P, or overlapping.

The functional implications of a motif will depend on the location within the protein and neighboring amino acids. Irrespective of the type of EAR motif sequence, the C-terminal region of the protein was found to be the most common location of EAR motifs (Figure 2). The middle region of the protein was found to be the second most frequent location of EAR motifs, with the N-terminal region being the least common. For the purposes of this review, the N-terminal region is defined as the first quarter of the protein, the C-terminal as the last quarter of the protein, and the remaining portion as the middle. When we break down the location of the EAR motif by sequence—that is LxLxL, DLNx(x)P and overlapping motifs—we see that the C-terminus is still the most common location of the EAR motif irrespective of the type of motif (Figure 2). It is worth noting that the total number of EAR motifs from each category varied (LxLxL = 98, DLNx(x)P = 33, overlapping = 12), and that the functional characterization of additional EAR motifs from each category could provide more insight into the location of these motifs.

Given that the EAR motif is most commonly identified in the C-terminus of a protein, it is not surprising that for 5 of the 7 biological functions we analyzed the EAR motif is most commonly identified in the C-terminal portion of the protein (Table 3). In two instances, however; the EAR motif was more often identified elsewhere in the protein: the middle region (Metabolism and homeostasis, 52%) and the N-terminal region (Hormonal pathway and signaling, 53%). Given that this review only analyzed 143 EAR motifs, the additional functional characterization of more EAR motif-containing transcriptional repressors would provide more insight into trends in localization across biological function.

Interestingly, of the 65 EAR motifs that contain a DLNx(x)P sequence (including motifs with overlapping LxLxL and DLNx(x)P sequences), only 5 were found outside of the C-terminal region. Though additional experimentation would be required, we hypothesize that there is a correlation between the DLNx(x)P motif and the C-terminal region of the protein.

This review did not explore the significance of the location of the EAR motif, since determining how its location affects transcriptional repression would require additional research. However, through the analysis of 119 EAR motif-containing proteins and the locations of 143 EAR motifs we are able to propose the C-terminal importance of DLNx(x)P EAR motifs in addition to the versatility of the LxLxL EAR motif across the entire protein sequence.

## 3. Molecular Functions of EAR Motif Containing Proteins

The EAR motif-containing proteins play key roles in diverse biological functions by negatively regulating genes involved in developmental, hormonal, and stress signaling pathways. The functionally characterized EAR repressors surveyed in this study can be classified into seven functional categories: (1) plant growth and morphology (Table S9), (2) metabolism and homeostasis (Table S10), (3) abiotic stress response (Table S11), (4) biotic stress response (Table S12), (5) hormonal pathway and signaling (Table S13), (6) fertility (Table S14), and (7) ripening (Table S15).

### 3.1. Growth and Morphology

A broad collection of 44 different EAR motif-containing proteins were found to affect plant growth and morphology across 13 different species (Table S9). For instance, the LxLxL EAR motif-containing repressor in SMXL7 affects the phenotype of the leaf, shoot, and shoot branching in *A. thaliana*, where the LxLxL motif reduces the target gene’s function via transcriptional repression [40]. In tomato (*Solanum lycopersicum*), SlEAD1 contains three overlapping LxLxL EAR motifs (LVLLLVLFL), which help to negatively regulate abscisic acid (ABA) response [37]. As abscisic acid is responsible for growth and development, the negative regulation of ABA in turn modulates root elongation of the plant [37]. The DLNx(x)P (DLNNLP) containing JAGGED1 repressor, which has been found in soybean (*Glycine max*), Arabidopsis (*A. thaliana*), and tomato (*S. lycopersicum*), also contributes to plant morphology [23]. Specifically, JAGGED1 promotes lateral organ development and affects fruit patterning in plants [23]. Also found in *A. thaliana*, the C2H2-type zinc finger protein AtZP1 acts as a transcriptional repressor that results in the negative regulation of the plant’s root hair growth [16]. This functionality is achieved through AtZP1’s LDLELRL (LxLxL) EAR motif, which is directly responsible for the gene’s prohibitory behavior [16]. AtWOX7, belonging to the WUSCHEL (WUS) transcription factor family, contains a LDLRL (LxLxL) motif, which is responsible for the development of the root system in *A. thaliana* [15].

### 3.2. Metabolism and Homeostasis

The maintenance of ion homeostasis is required for the survival of all plants. Homeostasis allows for the organization of defined cellular components, in which different biochemical processes can occur in their intended manner [49]. Membrane transport proteins play a significant role in maintaining chemical homeostasis [49]. We identified 27 different EAR motif-containing proteins whose primary function is to assist in modulating these processes in plants (Table S10).

The basic helix-loop-helix transcription factor bHLH11, containing an LxLxL type EAR motif, has been found to help regulate the homeostasis of Fe within various plants, including *A. thaliana*, *Zea mays*, and *Brassica rapa* [17]. Working in conjunction with TPL/TPR co-repressors, the expression of Fe deficiency-responsive genes is negatively regulated by bHLH11 with the help of the EAR motif [17]. A series of 21 R2R3-MYB repressors have been found to be involved in metabolism and chemical/biochemical regulation in potato (*Solanum tuberosum*) [13]. With the exception of MYB3-like S4, which contains a DLNx(x)P type of EAR motif, the other 20 of these StR2R3-MYB repressors contain LxLxL EAR motifs [13]. This collection of repressors collectively contributes to the potato plant’s ability to adapt in response to various environmental changes; specifically, by regulating biological, cellular and metabolic processes [13]. Similarly, in *Arabidopsis*, the AtWUS protein also helps maintain homeostasis within the plant at each stage of development [15]. AtWUS contains a LELRL EAR motif, which contributes to the maintenance of stem cell homeostasis in the shoot apical meristem [15].

In the pink periwinkle flowering plant (*Catharanthus roseus*), a collection of three repressors called ZCT1, ZCT2 and ZCT3 (all of which belong to the C2H2 type zinc-finger protein family) help to regulate secondary metabolism using their EAR motifs [12]. Of interest, two of these proteins (ZCT1 and ZCT2) contain overlapping LxLxL and DLNx(x)P C-terminal EAR motifs, LDLNLTP. The third (ZCT3) contains an N-terminal LALCL motif and a C-terminal DLNLP. Though more research is needed in order to determine the significance of the overlapping and multiple motifs in these ZTC proteins, this survey demonstrates the important role that EAR motifs play in plant metabolism and homeostasis.

### 3.3. Abiotic Stress Response

Given that plants are unable to move, they must be able to withstand the various abiotic stresses found in their environment such as drought, salinity, mineral toxicity and extreme temperatures such as cold, frost, and heat. In order to do so, plants have evolved regulatory pathways and networks which allow them to respond to these stresses in a timely manner [50]. These pathways increase the plant’s adaptive responses and ameliorate stress resistance, which in turn leads to crop improvement [50]. In certain plants, EAR motif-containing proteins play a large role in improving abiotic stress tolerance.

We identified 21 proteins involved in abiotic stress response across 6 different plant species (Table S11). Various transcription factor families including, but not limited to ERF, ZAT, DREB, and JAZ are represented within our abiotic stress response functional category [36,41,46,48]. Proteins that were designated to the abiotic stress response category were not limited to either type of EAR motif, with 18 of them containing the LxLxL motif, and the remaining 11 containing the DLNx(x)P type of EAR motif.

In fact, the StERF3 protein, which has been characterized in potato (*S. tuberosum*), contains two EAR motif sequences, LDLRL and LNLDLNFPP; therefore, it simultaneously contains both LxLxL and DLNx(x)P type EAR motifs [41]. While StERF3 has a broad range of functions and has been assigned to multiple functional categories, this ERF protein is thought to regulate salt response by interacting with histone deacetylases (HDACs) in order to block target genes from being transcriptionally activated [41]. Another example is the RAP2.1 protein of the DREB family [36]; found in *A. thaliana*, it contains a conserved DLNQIP EAR sequence and regulates plant responses to cold and drought stresses by interacting with DREB-type transcriptional activators [36]. The remaining EAR motif-containing abiotic stress response proteins that were included in our study can be found in Table 2.

### 3.4. Biotic Stress Response

In addition to abiotic stress responses, a strong biotic stress response system is crucial for plant survival. Within their environments, plants are exposed to a variety of biotic factors such as herbivore attacks, insects, and microbial pathogens, which can be severely detrimental to crop productivity and yield [51]. Plants rely on physical attributes such as a waxy cuticle and rigid cell walls in order to protect themselves from microbes and other pathogens [52]. As part of their immune system, plants have also been found to have a memory of previous infection, such that they can prime themselves should reinfection ever occur in the future [52]. As a result, it is crucial for plants to have a strong functioning biotic stress response where regulatory or transcriptional machinery can be activated to produce a targeted response against the specific stressor [51]. EAR motif-containing proteins play an instrumental role in contributing to plant defense and immunity in a variety of ways. For example, EAR motif-containing proteins have been found to help regulate cell death in response to pathogenic infections and induce wound response [12,48].

We identified 53 different EAR motif-containing proteins in 16 species, which function to protect plants against the biotic stress factors (Table S12). Like the abiotic stress response category, proteins belonging to the biotic stress response category also represent a diverse range of protein families. The EAR motif LELSLGLSL in TaJAZ1 increases resistance against powdery mildew in bread wheat (*Triticum aestivum*), which is a biotrophic fungal infection caused by *Blumeria graminis* [43]. The ability of TaJAZ1 to protect against powdery mildew is instrumental to crop success, as this disease is a major limitation for wheat production [43]. In soybean (*G. max*), the GmERF4 protein, which contains two EAR motifs (LDLNLAP and DLNHPP), builds resistance against *Phytophthora sojae,* a pathogenic infection resulting in stem and root rot [22]. Among the EAR motif-containing biotic stress response proteins identified (Table 2) the abiotic stress protein StERF3 was also found to have a biotic stress response in potato by protecting the crop against *Phytophthora infestans*, which is a fungal organism that causes potato blight disease [47]. These EAR motif-containing proteins play significant roles in building plant immunity against infections, and contribute largely to overall crop success and survival.

In corn (*Zea mays*), a series of LxLxL EAR motif-containing proteins belonging to the ZmJAZ family have been found to function in response to wounding [48]. Namely, ZmJAZ6 and ZmJAZ15 have been characterized as fast-acting, rapidly induced wound response proteins, both of which interact with TPL/TPR co-repressors [48]. Similarly, ZmJAZ23 also recruits TPL/TPR co-repressors in response to wounding; however, unlike ZmJAZ6 and ZmJAZ15, ZmJAZ23 is induced later on and is not as fast acting as its early-induced counterparts [48]. Of potential interest as well, two of these ZmJAZ family members, ZmJAZ26 and ZmJAZ34, contain two overlapping LxLxL motifs—LPLPLLL and LCLLLQL, respectively. Comparably, the EAR motif-containing (LDLSL, DLNLPP) transcriptional repressor, NbCD1, has been found to modulate hypersensitive cell death in tobacco in response to pathogenic infections [12]. As the largest functional category, an extensive understanding of these EAR motif-containing proteins as well as their functions is essential to the study of plant immunity.

Exploring the relationship between plant biotic stress responses and the pathogens that are responsible for these stresses could lead to novel insights into plant pathogen relationships. Plants have evolved to employ several defense strategies against pathogen invasion including physical barriers, chemical barriers, and mechanisms that rapidly clear infection and induce a defense response. Pathogens in turn have evolved to secrete effector proteins into host plant cells that share sequence, functional, and structural similarities with the host defense proteins. These pathogen effectors can act to modify host cell activities and gene expression to suppress host defense responses and ultimately create an environment that promotes growth, multiplication and transmission of the pathogen [53].

XopD is a type II effector from *Xanthomonas campestris* pv *vesicatoria (Xcv*), the causal agent of bacterial spot disease of tomato. XopD promotes pathogen multiplication by delaying the onset of leaf chlorosis and necrosis [54,55]. It is known to physically interact with transcriptional activators MYB30 and SIERF4 and suppress transcriptional activation of downstream defense- and senescence-associated genes in the parasitized host cells [55]. The XopD protein has a modular structure containing an N-terminal region, a helix-loop–helix DNA binding domain, two EAR motifs and a C-terminal cysteine protease domain with homology to a yeast ubiquitin-like modifier (SUMO) protease. Mutation analyses have revealed that both the EAR motifs and SUMO protease domain of XopD contribute to its virulence-associated functions [54,55].

Another study by Segonzac et al. (2017) demonstrated that a conserved EAR motif in another effector PopP2 from *Ralstonia solanacearum,* the causal agent of devastating bacterial wilt disease in a wide range of agronomically important host species [56], is required for its stability and avirulence function. The mutation of the EAR motif renders PopP2 unstable, affects the expression of defense genes and restricts bacterial growth [56], suggesting that the PopP2-mediated suppression of defense response and the host recognition ability of the pathogen are dependent on a functional EAR motif. HaRxL21 (RxL21), an RxLR effector from the oomycete pathogen *Hyaloperonospora arabidopsidis* causing Arabidopsis downy mildew, interacts with the plant co-repressor TPL via the EAR motif at the C-terminus of the effector, mimicking the host’s EAR-mediated transcriptional repression [57]. Similarly, Naked1 (Nkd1), a *Ustilago maydis* effector protein, interacts with the co-repressors TPL/TPRs and prevents the recruitment of a transcriptional repressor involved in jasmonate and auxin signaling, leading to suppression of PAMP-triggered ROS bursts and increased pathogen susceptibility [58]. These studies, while clearly establishing a role for EAR motif in plant-pathogen interactions, prompt us to speculate on a role for EAR motif in plant-microbe communications for symbiosis.

### 3.5. Hormonal Pathway/Signaling

Many EAR motif-containing proteins are involved in plant hormone (phytohormone) signaling pathways. Phytohormones are essential to plant survival as they help to carry out vital functions such as development, growth, and longevity, and help plants adapt to extreme environments [59]. In fact, phytohormones have been found to be associated with nearly all fundamental biological processes, which emphasizes the importance of understanding the pathways and networks that they are involved in, in order to ameliorate agronomic practices [59]. For example, salicylic acid (SA) plays an important role in defense and immunity as it contributes to pathogen recognition in plants [60].

In *A. thaliana* and *O. sativa* (rice), EAR motif-containing transcriptional repressors NIMIN1 and NRR have been identified to regulate SA signaling, respectively [12]. In apple (*Malus domestica*), MdMYB6 transcriptionally represses the accumulation of anthocyanin using its LSLSL EAR motif, by prohibiting the anthocyanin biosynthesis pathway without the assistance of any co-repressors [28]. In addition to the NIMIN1 and NRR motif-containing repressors, AtERF4 found in *A. thaliana* negatively regulates the expression of a gene involved in the expression of JA through its two EAR motifs: LELSL and LDLDLNLPP [31].

### 3.6. Fertility

EAR motif-containing repressors have also been found to be involved in developmental functions such as fertility within plants. The reproduction of germ line cells is particularly important in angiosperm reproduction as gametes are required in order to pass genetic information to the next generation [19].

Belonging to the DAZ family, which is a subfamily within the C2H2-type zinc finger protein group, DAZ1 and DAZ2 both contribute to sperm fertility and male germ cell division in *A. thaliana* [19]. Both of these repressors contain LDLRLGL and DLNx(x)P EAR motifs (DLNVP and DLNVPP, respectively) and work in conjunction with TPL/TPR co-repressors in order to promote fertility [19]. The MADS-box transcription factor AGL15 was also found to impact fertility in *Arabidopsis* [11]. With the help of AtSAP18 and TPL/TPR co-repressors, AGL15 directly represses target gene expression, which in turn promotes somatic embryogenesis in the plant [11]. Similarly, OsWOX1, belonging to the WUS homeobox transcription factor family, was found to regulate fertility in rice (*O. sativa*) [15]. OsWOX1’s ability to regulate fertility is a direct result of the LELTL EAR motif the repressor contains [15].

### 3.7. Ripening

Although EAR motif-containing transcriptional repressors contribute substantially to fertility, they also give rise to a broader range of functions in plants. In certain plants that produce fruit, the EAR motif helps to induce ripening. Understanding the process of fruit ripening, along with the functions of the genes involved in its regulation, is an essential component of improving crop development. According to Osorio et al. (2013), the process of ripening is a highly coordinated developmental process that involves thousands of genes, and progressively alters the firmness, flavor, color and taste of the fruit [61]. We discovered 13 EAR motif-containing proteins involved in the process of fruit ripening in both peach and tomato (Table S15).

For example, in peach (*Prunus persica*), the LxLxL motif-containing transcriptional repressor PpEIL1 helps to induce the fruit ripening process [33]. The EIL proteins (PpEIL1, PpEIL2, and PpEIL3) are thought to activate ethylene biosynthesis genes PpACO1 and PpACS1, which in turn ripens the peach fruit [33]. Likewise, SIERF.F12 in tomato *(S. lycopersicum*) has two separate EAR motif sites (LTLDLNLP and DLNEPP) and interacts with co-repressors TPL2 and histone deacetylases HDA1 and HDA2, in order to modulate the fruit ripening process [38].

## 4. Conclusions

This multispecies systematic review analyzed the biological functions and localizations of EAR motif-containing transcriptional repressors and provides insights into transcriptional repression in plants. Using a wide lens provided further insights into the functionality and processes of negative gene regulation for various developmental, metabolic, and hormonal processes in different species. By expanding the scope of our study to multiple species, we were able to investigate how evolution acts on the EAR motif-containing proteins in plants. There is supportive evidence that most of these proteins, along with their EAR motifs, are conserved across species and have evolved with these plants.

Despite its small size, the EAR motif has been found to be instrumental in regulating several biological functions and processes across numerous plant species. Though we grouped the proteins into 7 biological functional categories, some of our categories could be further broken down into more specific functions. For example, our growth and morphology category could be split into seed development, root growth, flowering, and leaf morphology.

Because positive gene regulation and transcriptional activation are studied so extensively, there remains so much more to discover about negative gene regulation, including EAR motif mediated transcriptional repression, and the role it plays in plant development and health. Furthermore, while EAR motifs are currently the predominant motif associated with transcriptional repression that has been identified in plants, additional research could lead to the identification of other novel repression motif-containing proteins.

## Figures and Tables

**Table 1:** Summary of EAR motif-containing proteins based on biological function. The number of proteins in each functional category, the number of plant species represented by those proteins, and the total number of LxLxL and DLNx(x)P EAR motifs in each functional category are provided.

Functional Category	Total Number of Proteins	Number of Plant Species	Plant Species	Number of LxLxL Sites	Number of DLNxxP Sites
Growth morphology	44	13	*Arabidopsis lyrata, Arabidopsis thaliana,* *Boechera stricta, Brassica rapa, Eutrema salsugineum, Glycine max, Malus domestica, Medicago truncatula, Oryza sativa, Prunus persica, Solanum lycopersicum,* *Sphagnum fallax, Triticum aestivum*	34	19
Metabolism homeostasis	27	4	*Arabidopsis thaliana*, *Catharanthus roseus*, *Solanum lycopersicum, Solanum tuberosum*	28	5
Abiotic stress response	21	6	*Arabidopsis thaliana, Ipomoea batatas, Oryza sativa*, *Petunia hybrida* cv. ‘Mitchell’, *Solanum tuberosum,* *Zea mays*	18	11
Biotic stress response	53	16	*Arabidopsis lyrata, Arabidopsis thaliana,* *Boechera stricta, Brassica rapa, Elaeis guineensis,* *Eutrema salsugineum, Glycine max, Ipomoea batatas,* *Nicotiana benthamiana, Oryza sativa, Pinus taeda, Prunus persica, Solanum tuberosum, Sphagnum fallax, Triticum aestivum, Zea mays*	55	14
Hormonal pathway signalling	25	8	*Arabidopsis thaliana*, *Malus domestica,* *Nicotiana tabacum*, *Oryza sativa, Solanum lycopersicum*, *Solanum tuberosum, Ustilago maydis*, *Zea mays*	26	7
Fertility	4	2	*Arabidopsis thaliana*, *Oryza sativa*	4	2
Ripening	13	2	*Prunus persica*, *Solanum lycopersicum*	10	8

**Table 2:** EAR motif-containing protein metadata table. This table summarizes the data from the fully characterized 119 EAR motif-containing proteins including their name, identifiers, motif sequence, starting amino acid position, location (N = N-terminal, M = middle, and C = C-terminal), the length of the protein, the plant species that the protein was identified in, and the biological function(s) of the protein (G = Plant growth/morphology, M = Metabolism/homeostasis, A = Abiotic stress response, B = Biotic stress response, H = Hormonal pathway/signaling, F = Fertility, and R = Ripening).

Gene Name	Gene Identifiers	EAR Motif Sequence	Start	Region	Size	Plant Species	Function	Ref.
488615	488615	LLLAL	463	C	506	*Arabidopsis lyrata*	G, B	[7]
AGL15	AT5G13790	LQLGL	214	C	271	*Arabidopsis thaliana*	F	[11]
AtERF4	AT3G15210	LELSL, LDLDLNLPP	127, 209	C, C	222	*Arabidopsis thaliana*	B, H	[12]
AtERF7	AT3G20310	DLNFPP	218	C	244	*Arabidopsis thaliana*	A, H	[12]
AtMYB4	AT4G38620	LNLEL	198	M	282	*Arabidopsis thaliana*	A	[12]
AtMYB73-like S22	PG0024983	LSLSL, LGLGL	205, 344	M, C	370	*Solanum tuberosum*	M, B	[13]
AtTCP20	AT3G27010	LELGL	252	C	314	*Arabidopsis thaliana*	G	[14]
AtWOX5	AT3G11260	LDLRL	175	C	182	*Arabidopsis thaliana*	G, H	[15]
AtWOX7	AT5G05770	LDLRL	181	C	122	*Arabidopsis thaliana*	G	[15]
AtWUS	AT2G17950	LELRL	287	C	292	*Arabidopsis thaliana*	M	[15]
AtZP1	AT3G50260	LDLELRL	193	C	204	*Arabidopsis thaliana*	G	[16]
AUX/IAA	AT4G14560	LRLGL	14	N	168	*Arabidopsis thaliana*	H	[12]
AZF2	AT3G19580	LALCL, DLNLP	71, 224	M, C	273	*Arabidopsis thaliana*	A	[12]
bHLH11	AT4G36060	LDLDL, LKLEL	208, 231	M, C	287	*Arabidopsis thaliana*	M	[17]
Bostr.26527s0463.1	Bostr.26527s0463.2	LLLAL	487	C	530	*Boechera stricta*	G, B	[7]
Bra013973	Bra013973	LLLAL	324	C	360	*Brassica rapa*	G, B	[7]
BZR1	AT1G75080	LELTL	325	C	336	*Arabidopsis thaliana*	G, H	[18]
DAZ1	AT2G17180	DLNVP, LDLRLGL	247, 264	C, C	270	*Arabidopsis thaliana*	G, F	[19]
DAZ2	AT4G35280	DLNVPP, LDLRLGL	258, 278	C, C	284	*Arabidopsis thaliana*	G, F	[19]
DEAR1	AT3G50260	DLNKLP	136	C	153	*Arabidopsis thaliana*	A, B	[20]
EgMYB4	KT778616	LNLEL	178	C	237	*Elaeis guineensis*	B	[21]
ERF3	AT1G50640	DLNFPP	200	C	225	*Arabidopsis thaliana*	A, B	[12]
GmERF4	ACE76905	LDLNLAP, DLNHPP	115, 212	M, C	222	*Glycine max*	B	[22]
GmJAGGED1	Glyma20g25000.1	DLNNLP	9	N	256	*Glycine max*	G	[23]
HSI2	AT2G30470	DLNSDP	729	C	790	*Arabidopsis thaliana*	H	[12]
IAA1	AT4G14560	LRLGL	14	N	168	*Arabidopsis thaliana*	H	[24]
IAA16	AT3G04730	LRLGL, LKLNL	9, 38	N, N	236	*Arabidopsis thaliana*	H	[24]
IAA19	AT2G33310	LRLGL	9	N	197	*Arabidopsis thaliana*	H	[24]
IAA27	AT4G29080	LRLGL	45	N	305	*Arabidopsis thaliana*	H	[24]
IAA28	AT5G25890	LELRL	7	N	175	*Arabidopsis thaliana*	G, H	[24]
IAA3	AT1G04240	LLLLL, LRLGL	23, 112	N, M	289	*Arabidopsis thaliana*	H	[24]
IAA4	AT5G43700	LRLGL	18	N	186	*Arabidopsis thaliana*	H	[24]
IAA5	AT1G15580	LRLGL	15	N	163	*Arabidopsis thaliana*	H	[24]
IAA6	AT1G52830	LRLGL	13	N	189	*Arabidopsis thaliana*	H	[24]
IAA7	AT3G23050	LCLGL, LMLNL	13, 44	N, N	287	*Arabidopsis thaliana*	G, H	[8]
IbERF4	ARS72978	LELDL, LNLDLNLAP	112, 215	M, C	227	*Ipomoea batatas*	A, B	[25]
JAZ8	AT1G30135	LELRL	9	N	131	*Arabidopsis thaliana*	H	[26]
Jsi1	UMAG_01236	DLNELP	39	N	641	*Ustilago maydis*	H	[27]
MdMYB6	MDP0000198015	LSLSL	217	M	312	*Malus domestica*	G, H	[28]
MtRSD	AFQ94047	LDLELRL	129	C	151	*Medicago truncatula*	G	[29]
MYB101-like S18	PG0013897	LSLTL	237	M	476	*Solanum tuberosum*	M, B	[13]
MYB101-like S18	PG0028949	LPLTL	200	M	484	*Solanum tuberosum*	M, B	[13]
MYB108-like S20	PG0008761	LILEL	89	M	297	*Solanum tuberosum*	M, B	[13]
MYB108-like S20	PG0004612	LILQL	68	M	230	*Solanum tuberosum*	M, B	[13]
MYB108-like S20	PG0027157	LILEL	104	M	324	*Solanum tuberosum*	M, B	[13]
MYB108-like S20	PG1004611	LILQL	68	M	234	*Solanum tuberosum*	M, B	[13]
MYB108-like S20	PG2004611	LILQL	100	M	240	*Solanum tuberosum*	M, B	[13]
MYB15-like	PG0015087	LILNL	77	M	243	*Solanum tuberosum*	M, B	[13]
MYB15-like S2	PG0020071	LDLSL, LMLEL	220, 251	C, C	258	*Solanum tuberosum*	M, B	[13]
MYB17-like S9	PG0000027	LQLLL	278	C	309	*Solanum tuberosum*	M, B	[13]
MYB17-like S9	PG0021654	LQLLL	274	C	319	*Solanum tuberosum*	M, B	[13]
MYB3-like S4	PG0030548	DLNSLP	173	C	178	*Solanum tuberosum*	M, B	[13]
MYB32-like S4	PG0006176	LNLEL	188	M	254	*Solanum tuberosum*	M, B	[13]
MYB4-like S4	PG0013215	LNLEL	189	M	268	*Solanum tuberosum*	M, B	[13]
MYB41-like	PG0020012	LELYL	232	C	243	*Solanum tuberosum*	M, B	[13]
MYB44-like S22	PG0003316	LCLSL	206	M	320	*Solanum tuberosum*	M, B	[13]
MYB48-like	PG0015536	LVLEL	72	M	218	*Solanum tuberosum*	M, B	[13]
MYB62-like S20	PG0005641	LILEL	88	M	278	*Solanum tuberosum*	M, B	[13]
MYB62-like S20	PG0014550	LILEL	84	M	273	*Solanum tuberosum*	M, B	[13]
MYB70	Solyc04g078420	LRLSL	215	M	309	*Solanum lycopersicum*	R	[30]
MYB70-like S22	PG0044858	LSLSL	227	C	302	*Solanum tuberosum*	M, B	[13]
NbCD1	BAD99476	LDLSL, LDLNLPP	122, 221	M, C	231	*Nicotiana benthamiana*	B	[12]
NIMIN1	AT1G02450	LDLNLAL	136	C	142	*Arabidopsis thaliana*	B, H	[12]
NRR	Os01g0130200	DLNVEP	107	C	127	*Oryza sativa*	B, H	[12]
NtERF3	BAJ72664	LELDL, LDLNLAP	114, 215	M, C	225	*Nicotiana benthamiana*	B	[31]
OsERF3	BAB03248	LDLDL, DLNRPP	129, 226	M, C	235	*Oryza sativa*	A, B	[32]
OsWOX1	Os04t0663600-01	LELTL	270	C	289	*Oryza sativa*	F	[15]
OsWOX9	CAJ84144	LELRL	191	C	200	*Oryza sativa*	G	[15]
PpEIL1	ABK35085	LKLGL	215	M	601	*Prunus persica*	R	[33]
PpERF3b	Ppa010804m	DLNLPP	201	C	235	*Prunus persica*	G, B	[34]
PtMYB14	DQ399056	LNLDL	164	C	192	*Pinus taeda*	B	[35]
Q	UPQ43659	LDLDL	292	M	447	*Triticum aestivum*	G	[9]
RAP2.1	AT1G46768	DLNQIP	143	C	153	*Arabidopsis thaliana*	A	[36]
SlEAD1	Solyc12g099500	LVLLLVLFL	97	C	119	*Solanum lycopersicum*	G, H	[37]
SlERF.F12	Solyc02g077840	LTLDLNLP, DLNEPP	79, 145	M, C	154	*Solanum lycopersicum*	R	[38]
SlERF10-1	Solyc10g006130.1.1	DLNFPP	197	C	221	*Solanum lycopersicum*	G	[10]
SlERF10-2	Solyc10g009110.1.1	LDLSL, LNLDLNFPP	125, 210	M, C	222	*Solanum lycopersicum*	G	[10]
SlERF12-1	Solyc12g005960.1.1	DLNFPP	175	C	193	*Solanum lycopersicum*	G	[10]
SlERF2-10	Solyc02g093130.1.1	DLNLKP	118	C	133	*Solanum lycopersicum*	G, M	[10]
SlERF2-6	Solyc02g077840.1.1	LPLLL	75	C	99	*Solanum lycopersicum*	G, R	[10]
SlERF3-16	Solyc03g117230.1.1	LDLNL	243	C	252	*Solanum lycopersicum*	G, R	[10]
SlERF3-4	Solyc03g006320.1.1	LDLSL, DLNLLP	119, 207	M, C	216	*Solanum lycopersicum*	G, R	[10]
SlERF36	NP_001355161	DLNFPP	197	C	221	*Solanum lycopersicum*	G	[39]
SlERF4-1	Solyc04g007180.1.1	LDLEL	272	C	350	*Solanum lycopersicum*	G, R	[10]
SlERF4-10	Solyc04g078640.1.1	DLNEYP	134	C	148	*Solanum lycopersicum*	G, R	[10]
SlERF4-11	Solyc04g080910.1.1	DLNFPP	240	C	249	*Solanum lycopersicum*	G, R	[10]
SlERF5-8	Solyc05g052030.1.1	LTLEL	8	N	201	*Solanum lycopersicum*	G, R	[10]
SlERF7-2	Solyc07g049490.1.1	DLNLPP	174	C	198	*Solanum lycopersicum*	G, R	[10]
SlERF7-3	Solyc07g053740.1.1	LELDL, LDLDLNLAP	121, 213	M, C	225	*Solanum lycopersicum*	G, R	[10]
SlERF7-5	Solyc07g054220.1.1	DLNLP	214	C	240	*Solanum lycopersicum*	G, R	[10]
SlERF9-1	Solyc09g009240.1.1	DLNFLP	88	M	202	*Solanum lycopersicum*	G	[10]
SlERF9-10	Solyc09g091950.1.1	LGLFL	123	M	419	*Solanum lycopersicum*	G	[10]
SMXL7	AT2G29970	LDLNLP	854	C	1002	*Arabidopsis thaliana*	G	[40]
Sphfalx0198s0025.1	Sphfalx0198s0025.2	LLLSL	75	C	88	*Sphagnum fallax*	G, B	[7]
Sphfalx0442s0002.1	Sphfalx0442s0002.2	LLLSL	396	C	433	*Sphagnum fallax*	G, B	[7]
StERF3	ABK96798	LDLRL, LNLDLNFPP	126, 211	M, C	223	*Solanum tuberosum*	A, B, H	[41]
StZFP1	ABK78777	LALCL, DLNMP	50, 223	N, C	266	*Solanum tuberosum*	A, B	[42]
TaJAZ1	QBQ83006	LELSLGLSL	5	N	476	*Triticum aestivum*	B	[43]
TaWOX9	EMS65007	LELRL	140	C	173	*Triticum aestivum*	G	[15]
Thhalv10015535m	Thhalv10015535m	LLLAL	454	C	549	*Eutrema salsugineum*	G, B	[7]
TIE1	AT4G28840	LDLELRL	187	C	193	*Arabidopsis thaliana*	G	[44]
ZAT10	AT1G27730	DLNIPP	190	C	227	*Arabidopsis thaliana*	A	[12]
ZAT12	AT5G59820	LDLSL, LNLKLEL	141, 151	C, C	162	*Arabidopsis thaliana*	A, B	[12]
ZAT4	AT2G45120	DLNLP	295	C	314	*Arabidopsis thaliana*	G	[45]
ZAT7	AT3G46090	LDLDL	144	C	168	*Arabidopsis thaliana*	A, B	[46]
ZAT9	AT3G60580	DLNLP	271	C	288	*Arabidopsis thaliana*	G	[45]
ZCT1	AJ632082	LDLNLTP	153	C	178	*Catharanthus roseus*	M	[12]
ZCT2	AJ632083	LDLNLTP	164	C	190	*Catharanthus roseus*	M	[12]
ZCT3	AJ632084	LALCL, DLNLP	62, 216	N, C	259	*Catharanthus roseus*	M	[12]
ZFT1	AB186899	LALCL, DLNIP	53, 211	N, C	253	*Nicotiana tabacum*	H	[12]
ZmCLA4	GRMZM2G135019	LVLEL	409	C	413	*Zea mays*	H	[47]
ZmJAZ15	GRMZM2G173596	LALEL	156	C	160	*Zea mays*	A, B	[48]
ZmJAZ23	GRMZM2G143402	LSLSL	43	N	230	*Zea mays*	A, B	[48]
ZmJAZ26	GRMZM2G114681	LPLPLLL	25	N	410	*Zea mays*	A, B	[48]
ZmJAZ34	Zm00001d041045	LCLLLQL	178	M	206	*Zea mays*	A, B	[48]
ZmJAZ4	GRMZM2G024680	LALRL	212	C	216	*Zea mays*	A, B	[48]
ZmJAZ5	GRMZM2G145412	LKLAL	178	C	182	*Zea mays*	A, B	[48]
ZmJAZ6	GRMZM2G145458	LTLTL	152	C	162	*Zea mays*	A, B	[48]
ZPT2-3	DD138888	DLNIP	210	C	253	*Petunia x hybrida*	A	[12]

**Table 3:** EAR motif localization compared to biological function. Distribution of the EAR motif locations in the 119 proteins in this study arranged by biological function and location within the protein (N = N-terminal, M = middle, C = C-terminal). EAR motifs are grouped by consensus sequences for DLNx(x)P, LxLxL, and overlapping motifs in addition to common or important variations of those motifs. Values expressing the percentage of EAR motifs in a given protein location per biological function are also expressed at the bottom of the table.

	Plant Growth/Morphology			Metabolism/Homeostasis			Abiotic Stress Response			Biotic Stress Response			Hormonal Pathway/Signaling			Fertility			Ripening		
Location	N	M	C	N	M	C	N	M	C	N	M	C	N	M	C	N	M	C	N	M	C
DLNxP			4			1			3			1			1			1			1
DLNxxP	1	1	10			2			6			7	1		3			1			5
LDLNLP			1																		
LDLNLxP						2					1	2									
LxLDLNLP																				1	
LxLDLNxxP			2						2			3			1						1
LxLxL	4	6	15	1	16	9	2	5	6	2	20	22	15	3	4			2	1	4	3
LxLxLxL							1	1	1	1	1	2			1			2			
LxLxLxLxL			1							1					1						
Motifs/location	5	7	38	1	16	14	3	6	18	4	22	37	16	3	11	0	0	6	1	5	10
Total motifs	50			31			27			63			30			6			16		
%motifs/location	10	14	76	3	52	45	11	22	67	6	35	59	53	10	37	0	0	100	6	31	63

## Supplementary Materials

The following supporting information can be downloaded at: https://www.mdpi.com/article/10.3390/genes14020270/s1, Table S1: Complete version of the metadata table showing EAR motif containing proteins; Table S2: DLNxP EAR motifs and DLNxP overlapping motifs; Table S3: DLNxxP EAR motifs and DLNxxP overlapping motifs; Table S4: DLNxxxP EAR motifs and DLNxxxP overlapping motifs; Table S5: DLNxxxxP EAR motifs; Table S6: LxLxL EAR motifs and LxLxL overlapping motifs; Table S7: LxLxLxL EAR motifs and LxLxLxL overlapping motifs; Table S8: LxLxLxLxL EAR motifs; Table S9: EAR motifs associated with plant growth and morphology; Table S10: EAR motifs associated with metabolism and homeostasis; Table S11: EAR motifs associated with abiotic stress response; Table S12: EAR motifs associated with biotic stress response; Table S13: EAR motifs associated with hormonal pathway and signaling; Table S14: EAR motifs associated with fertility; Table S15: EAR motifs associated with ripening.
